# Analysis of the Interaction Between the Attenuated HSV-1 Strain M6 and Macrophages Indicates Its Potential as an Effective Vaccine Immunogen

**DOI:** 10.3390/v17030392

**Published:** 2025-03-10

**Authors:** Zhenxiao Zhang, Xiaohong Ren, Ying Zhang, Jingjing Zhang, Xinghang Li, Fengyuan Zeng, Rong Yue, Qi Li, Haobo Zhang, Danjing Ma, Yuansheng Liao, Yun Liao, Dandan Li, Li Yu, Guorun Jiang, Heng Zhao, Huiwen Zheng, Heng Li, Xin Zhao, Longding Liu, Qihan Li

**Affiliations:** 1 Institute of Medical Biology, Chinese Academy of Medical Sciences & Peking Union Medical College, Kunming 650118, China; zzxs2021018027@imbcams.com.cn (Z.Z.); s2023018014@pumc.edu.cn (X.R.); zhangy@imbcams.com.cn (Y.Z.);; 2 Key Laboratory of Systemic Innovative Research on Virus Vaccines, Kunming 650118, China

**Keywords:** herpes simplex virus type 1, attenuated strain M6, attenuated vaccine, macrophages, immune response

## Abstract

Herpes simplex virus type 1 (HSV-1) is a very concerning pathogen due to its ability to persist in the host’s nervous system and continuously interfere with the immune system, which complicates treatment. Therefore, the development of an effective HSV-1 vaccine is crucial. In this study, we focused on an HSV-1 mutant strain, M6, which includes several deleted genes associated with viral infection virulence and latent infection function, and explored its infection of macrophages and immunological characteristics. The study found that both the attenuated strain M6 and the wild-type strain infect macrophages through the binding of the gD protein to the HVEM receptor on the macrophage surface. Compared to the wild-type strain, the attenuated M6 strain induced a milder immune response, characterized by the lower expression of immune signaling molecules and inflammatory cytokine levels. Upon reintroducing macrophages infected with the two strains into mice, the M6 strain induced lower levels of inflammatory cytokines and higher levels of chemokines in spleen cells and also slightly lower humoral and cellular immune responses than the wild-type strain. Further histopathological analysis revealed that mice in the attenuated M6 group showed more stable body weight changes and milder pathological damage in immune organs such as the liver, spleen, and lymph nodes. In conclusion, the attenuated M6 strain exhibits good immunogenicity and mild pathological side effects, suggesting its potential as an effective immunogen.

## 1. Introduction

Herpes simplex virus type 1 (HSV-1) is a pathogen that can be transmitted through contact and cause a variety of clinical manifestations, including oral herpes and genital herpes [1,2,3]. This virus has garnered widespread attention not only because of the widespread transmission of its diseases and their severe impact on individual quality of life [4,5,6] but also because of its complex pathological features. HSV-1 is capable of latent infection in the nervous system, severely interfering with the immune system [7,8] and making the associated diseases difficult to control and prevent [9,10]. Existing pathogenic studies have shown that the viral membrane carries various glycoproteins, which can bind to different cellular receptors [11,12,13,14]. Among these, the binding of gD protein to the HVEM receptor enables the virus to infect various cells, including dendritic cells and macrophages, which are involved in the innate immune response and play a key role in antigen recognition and presentation [15]. Therefore, in previous vaccine studies, relying solely on in vitro cell culture assays to detect neutralizing antibodies has made it difficult to develop practically significant vaccine products [16]. This limitation has driven research into the use of attenuated live vaccines in different forms. However, the basic premise of this research is to understand how an HSV-1 live vaccine with attenuated properties can maintain a comprehensive antigenic stimulation effect while minimizing interference with cells that have critical innate immune functions. This presents two main challenges. First, the attenuated strain must ensure that the major components related to infection virulence have been removed, which is a highly challenging task [17,18]. For example, removing the gD protein can effectively reduce the impact of the vaccine virus on immune cells, but this will also prevent the vaccine-induced immune response from blocking the virus’s infection through the interaction of the gD protein with the HVEM receptor. Therefore, maintaining key antigenic proteins in the vaccine strain while balancing its immune efficacy and pathological side effects during in vivo proliferation is a key aspect of vaccine research. Second, the cost–benefit ratio between the immune efficacy and pathological side effects of the attenuated strain needs to be analyzed and assessed [19,20]. The research team led by XU X used CRISPR/Cas9 technology to prepare an HSV-1 mutant, M6, which deleted genes UL7, UL41, LAT, US3, US11, and US12, all of which are related to viral transcriptional proliferation, infection virulence, and latent infection, and showed significant immunoprotective effects [21,22,23]. However, as a potential vaccine immunogen, further exploration of its immunogenicity and the mechanism behind its possible infective effects on immune cells remains necessary. These studies are not only critical in understanding the application mechanism behind this attenuated strain but also help us better understand the mechanisms by which HSV-1 infects innate immune cells and those behind its subsequent effects. The research team led by ZHANG J comprehensively analyzed the effects of M6 and the wild-type strain on dendritic cells and confirmed that the attenuated M6 strain exhibited reduced interference with dendritic cell function, demonstrating good immunogenicity [24]. In this study, we selected macrophages, which also play a significant role in innate immune responses, as the subject of investigation, and explored the effects and differences between the attenuated M6 strain and the wild-type strain on these cells. This study provides clear evidence for a better understanding of the mechanistic role of macrophages in the HSV-1 infection process, as well as the immune efficacy and pathological side effects of the attenuated M6 strain, laying a solid foundation for the in-depth study of M6.

## 2. Materials and Methods

### 2.1. Ethics Statement

All animal experiments were conducted in strict accordance with ethical and humane guidelines. Female BALB/c mice, aged 8 weeks, were selected for this study. These mice were housed in a specific pathogen-free facility at the Institute of Medical Biology, Chinese Academy of Medical Sciences (IMB, CAMS), under a 12 h light/dark cycle, with free access to food and water, and were cared for by a professional team. All mouse-related experiments were conducted in accordance with the “Guidelines for the Care and Use of Laboratory Animals” published by the U.S. National Institutes of Health and were reviewed and approved by the Institutional Animal Care and Use Committee (IACUC) of IMB, CAMS (Approval No: DWSP202312015).

### 2.2. Cell Lines

The Vero African green monkey kidney cell line (ATCC, Manassas, VA, USA) was cultured in MEM medium (Thermo Fisher Scientific, Waltham, MA, USA) containing 10% penicillin (100 U/mL), streptomycin (100 mg/mL), and 10% Newborn Bovine Serum (NBS; Minhai Biology, Gansu, China). The susceptible cells for HSV-1 wild-type strain 8F were infected with the virus, and the medium was replaced with MEM containing 2% NBS.

The KMB17 cell line (IMB, CAMS, Yunnan, China) was cultured in MEM medium containing 10% penicillin (100 U/mL), streptomycin (100 mg/mL), and 10% Fetal Bovine Serum (FBS; HyClone, GE Healthcare, Chicago, IL, USA). These cells were susceptible to HSV-1 mutant strain M6, and after virus infection, the medium was replaced with MEM containing 2% FBS.

The RAW264.7 mouse macrophage cell line (ATCC, Rockefeller, Gaithersburg, MD, USA) was cultured in DMEM medium (Thermo Fisher Scientific, Waltham, MA, USA) containing 10% penicillin (100 U/mL), streptomycin (100 mg/mL), and 10% FBS. After virus infection, the medium was replaced with DMEM containing 2% FBS.

### 2.3. Virus

HSV-1 Wild-Type Strain 8F is stored at IMB, CAMS. HSV-1 Mutant Strain M6 is stored at IMB, CAMS. The virus titer was determined using the method described below [25].

The titer of HSV-1 8F was determined using the standard virus titration method in Vero cells. The virus sample was serially diluted 10-fold in serum-free MEM, and 100 μL of each dilution was added to a 96-well plate, with 8 replicates for each dilution. Each well in the 96-well plate contained 3 × 10^5^ cells/mL of Vero cells. After incubating the plate at 37 °C with 5% CO_2_ for 7 days, the cytopathic effect (CPE) was evaluated under an inverted microscope (Nikon, Tokyo, Japan).

The titer of HSV-1 M6 was determined using the standard virus titration method in KMB17 cells. The virus sample was serially diluted 10-fold in serum-free DMEM, and 100 μL of each dilution was added to a 96-well plate, with 8 replicates for each dilution. Each well in the 96-well plate contained 3 × 10^5^ cells/mL of KMB17 cells. After incubating the plate at 37 °C with 5% CO_2_ for 7 days, the cytopathic effect (CPE) was evaluated under an inverted microscope (Nikon, Tokyo, Japan).

All virus-related experiments were conducted under Biosafety Level (BSL) 2 conditions, and the experiments were approved by the Biosafety Office of the Institute of Medical Biology, Chinese Academy of Medical Sciences (SWAQ20221201).

### 2.4. Antibodies

Rabbit anti-HSV1 gD antibody (Bioss, Beijing, China), with a volume of 100 μL and a concentration of 1 mg/mL, was used to treat HSV-1 wild-type strain 8F or HSV-1 mutant strain M6, to bind with the HSV-1 gD membrane protein, preventing gD from binding to the HVEM receptor on host cells. This simulates the scenario of gD blockade during the infection process of HSV-1 on RAW264.7 macrophages.

Rabbit anti-TNFRSF14 antibody (Bioss, Beijing, China), with a volume of 200 μL and a concentration of 1 mg/mL, was used to treat RAW264.7 macrophages, binding to the HVEM receptor on the macrophages, thereby preventing the HVEM receptor from binding to the HSV-1 gD membrane protein. This simulates the scenario of HVEM blockade during the infection process of HSV-1 on RAW264.7 macrophages.

### 2.5. Mouse Experiment Design

BALB/c mice were randomly divided into three groups. In the first group, macrophages were infected with HSV-1 8F for 24 h and then re-infused into the mice via the tail vein at a concentration of 3 × 10^5^/100 μL. In the second group, macrophages were infected with HSV-1 M6 for 24 h and then re-infused into the mice via the tail vein at a concentration of 3 × 10^5^/100 μL. In the third group, macrophages were not treated and were re-infused into the mice via the tail vein at a concentration of 3 × 10^5^/100 μL. These macrophages were sorted from the mice’s spleens using flow cytometry. The mice were observed daily and provided with a comfortable living environment. After a set period post-adoptive transfer, spleens were collected for qRT-PCR to measure changes in cytokine levels, blood samples were taken for neutralizing antibody detection, splenic lymphocytes were isolated for ELISpot assays, and some tissues were collected for histopathological analysis.

### 2.6. Viral Infection of RAW264.7 Macrophages

RAW264.7 macrophages were randomly divided into three groups. In the first group, RAW264.7 cells were infected with HSV-1 8F; in the second group, RAW264.7 cells were treated with HVEM-specific antibody before being infected with HSV-1 8F; in the third group, RAW264.7 cells were infected with HSV-1 8F that had been pre-treated with gD-specific antibody. Virus titers were measured at 12, 24, 36, and 48 h, and cytokine levels were assessed.

For the virus infection experiments, the MOI (Multiplicity of Infection) was set to 0.1, and the same infection experiment was conducted using the mutant strain HSV-1 M6.

### 2.7. Proliferation Kinetics Assay

Virus samples were collected within 72 h, and the virus titers of these samples were measured. The proliferation curve was plotted using GraphPad Prism 9.51.

### 2.8. Cytokine Analysis

We used qRT-PCR to measure cytokine expression in cells and tissues. Total RNA was extracted by using TRIzol (TaKaRa, Beijing, China). The specific primers used in the experiment are listed in Supplementary Table S1. Quantitative reverse transcription PCR (qRT-PCR) was performed using the One Step TB Green PrimeScript PLUS RT-PCR Kit (TaKaRa, Beijing, China) under the following cycling conditions: 42 °C for 5 min, 95 °C for 5 s, and 60 °C for 20 s, repeated for 40 cycles. Mouse glyceraldehyde 3-phosphate dehydrogenase (GAPDH) was used as the reference gene for normalization. Gene expression was reported as the fold change relative to the levels in the blank control group or uninfected virus samples used for calibration (2^−ΔΔCt^).

### 2.9. Neutralization Assay

We followed the standard procedure for neutralizing antibody measurement. The virus sample was diluted to 2 × 10^3^ CCID50/mL, and 50 μL of the virus dilution was added to each well of a 96-well plate. The serum was inactivated at 56 °C for 30 min. The serum was then serially diluted starting at a 1:2 ratio, with five successive two-fold dilutions (1:2, 1:4, 1:8, 1:16, and 1:32), and 50 μL of each serum dilution was added to the 96-well plate. The plate was incubated at 37 °C for 2 h. After dilution, cells were added at 2 × 10^4^ cells/100 μL per well to the 96-well plate. The plate was incubated at 37 °C with 5% CO_2_ for 7 days, and the cytopathic effect (CPE) was observed to determine the neutralizing antibody titer for each serum sample. A cell control group and a virus control group were included. The cell control group served as a negative control, with no expected CPE. The virus control group used the virus stock, which was diluted to 10^−1^ and 10^−2^, and 50 μL of each dilution was added to the 96-well plate along with 100 μL of the cell suspension. The expected results were that the cell control group would show no CPE, the undiluted virus would cause complete CPE, the 10^−1^ dilution would cause partial CPE, and the 10^−2^ dilution would cause no CPE. If the control group results met these criteria, then the neutralizing antibody assay would be considered valid.

### 2.10. Cell Sorting

CD11b^+^ and F4/80^+^ mouse splenic macrophages were sorted at the Kunming Institute of Zoology, Chinese Academy of Sciences (KIZ, CAS), using a high-speed multi-channel cell sorter (Beckman MoFlo Astrios EQs, Beckman, Brea, CA, USA). The sorted cells were cultured in Roswell Park Memorial Institute (RPMI) 1640 medium (Gibco, Grand Island, NY, USA) containing 20% FBS, under conditions of 37 °C and 5% CO_2_.

### 2.11. ELISpot Assay

Under sterile conditions, the spleens of mice were isolated and single-cell suspensions were prepared. Lymphocytes were isolated using a lymphocyte separation medium (Dako BioScience, Beijing, China). IFN-γ and IL-4 were detected according to the instructions of the mouse IFN-γ (or IL-4) ELISpot kit (MABTECH Inc., Cincinnati, OH, USA). After adding 10 mg of the stimulant (peptide: gB498-505, SSIEFARL) (GenScript Biotech, Shanghai, China) to the plate, spleen lymphocytes were seeded, with positive controls (PHA addition), negative controls (no stimulant), and blank controls (only medium) set up accordingly. The cells were then incubated at 37 °C for 30 h. Afterward, the cells and medium were removed and the plate was developed. Colored spots were counted using an automated ELISpot reader (CTL, Cleveland, OH, USA).

### 2.12. Statistical Analysis

The experiments were performed independently three times, and all data are presented as means with their standard errors. Two-way analysis of variance (ANOVA) (GraphPad Prism 9.51; GraphPad Software, San Diego, CA, USA) was used to analyze the significant differences between groups. A *p*-value < 0.05 was considered statistically significant.

## 3. Results

### 3.1. HSV-1 M6 Attenuated Strain Infects Macrophages Primarily via Its Membrane gD Protein Binding to HVEM

**Figure 1:**
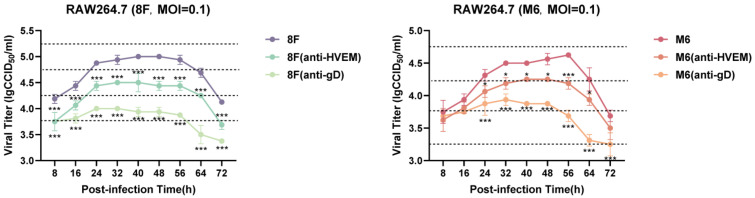
Proliferation kinetics curves of RAW264.7 macrophages infected with HSV-1 wild-type strain and attenuated strain M6 upon gD or HVEM blocking. Data are derived from three independent replicate experiments. The data are presented as the mean ± standard deviation of the three experiments (* *p* < 0.05, *** *p* < 0.001).

Previous studies have shown that the interaction between the HSV-1 membrane glycoprotein gD and HVEM determines the virus’s tendency to infect specific innate immune cells [26]. In this study, we first analyzed the infection patterns of the HSV-1 attenuated strain and wild-type strain in macrophages. Using RAW264.7 macrophages, which possess the HVEM-like receptor in mice [27], we conducted a blocking analysis with antibodies against gD protein and HVEM (Figure 1). We observed that although the use of a specific anti-HVEM antibody significantly reduced the viral infectivity, the effect of this antibody did not completely block viral replication. However, when using an anti-gD antibody, viral replication was nearly completely abolished, indicating that the binding of gD protein to the HVEM receptor is the primary pathway for the viral infection of macrophages.

### 3.2. HSV-1 Attenuated Strain Induces Weaker NF-κB Signaling Response in Macrophages

**Figure 2:**
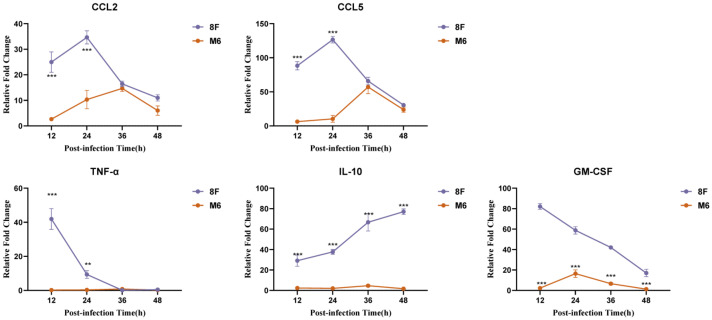
The binding of gD protein from HSV-1 wild-type strain and attenuated strain M6 to HVEM induced changes in the mRNA transcription kinetics of immune signaling molecules associated with NF-κB signaling responses. Data are presented as the mean ± standard deviation from three independent experiments (** *p* < 0.01, *** *p* < 0.001).

Laurent Derré’s team demonstrated that HVEM, as a member of the TNF receptor family, can activate the intracellular NF-κB signaling pathway upon binding with its two ligands, BTLA and CD160 [28], thereby mediating immune signaling functions [29,30]. This provides a foundation for analyzing the effects of the HSV-1 wild-type and attenuated strains on macrophages. By monitoring the mRNA transcription kinetics of immune signaling molecules related to NF-κB signaling, we observed the signaling response dynamics of macrophages after the binding of the gD protein from wild-type strain 8F and attenuated strain M6 to the HVEM receptor. The results showed that the expression levels of downstream effector molecules of the NF-κB pathway, such as CCL2, CCL5, and TNF-α, were significantly lower in the M6 group compared to the wild-type strain 8F (Figure 2). This indicates that the attenuated strain M6 induces a weaker NF-κB signaling response when infecting macrophages through the gD-HVEM pathway.

### 3.3. Compared to the Wild-Type Strain, the Attenuated Strain M6 Exhibits Similar Proliferation Dynamics in Macrophages but Induces Lower Levels of Immune Signaling Molecules

**Figure 3:**
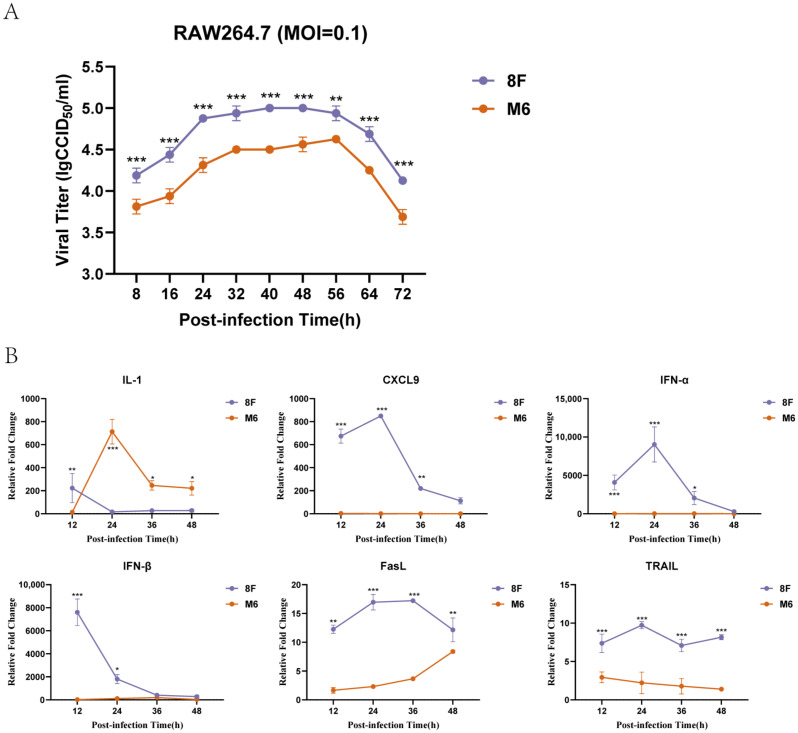
Differences in proliferation kinetics and induced immune signaling molecule levels between wild-type HSV-1 and attenuated M6 strain infection in RAW264.7 macrophages. (**A**) Proliferation kinetics curve. (**B**) Changes in levels of certain immune signaling molecules. The relative expression levels of cytokines in RAW264.7 macrophages were standardized to the levels of the blank control group using the comparative Ct (ΔΔCt) method. Data are presented as the mean ± standard deviation from three independent experiments (* *p* < 0.05, ** *p* < 0.01, *** *p* < 0.001).

Previous experiments indicated that the wild-type and attenuated strains share similarities in the process of adsorbing to macrophages. We further examined the proliferation dynamics of the two strains in macrophages, and the results showed that the proliferation dynamics of attenuated strain M6 were similar to those of the wild-type strain 8F (Figure 3A). Next, we investigated the effects of these two strains on the macrophage immune response, focusing on the expression changes in several key immune signaling molecules, such as IL-1, CXCL9, and IFN-α. The results demonstrated that at different time points after infection, the wild-type strain 8F induced a stronger immune response, as evidenced by the significant upregulation of immune signaling molecule expression (Figure 3B). In contrast, the attenuated strain M6 induced a weaker immune response, with significantly lower expression levels compared to 8F. These findings suggest that while the two strains exhibit similar proliferation dynamics, they induce significantly different levels of immune signaling molecules.

### 3.4. Infection with the Attenuated Strain M6 Induces Host-Specific Immune Responses in Macrophages

**Figure 4:**
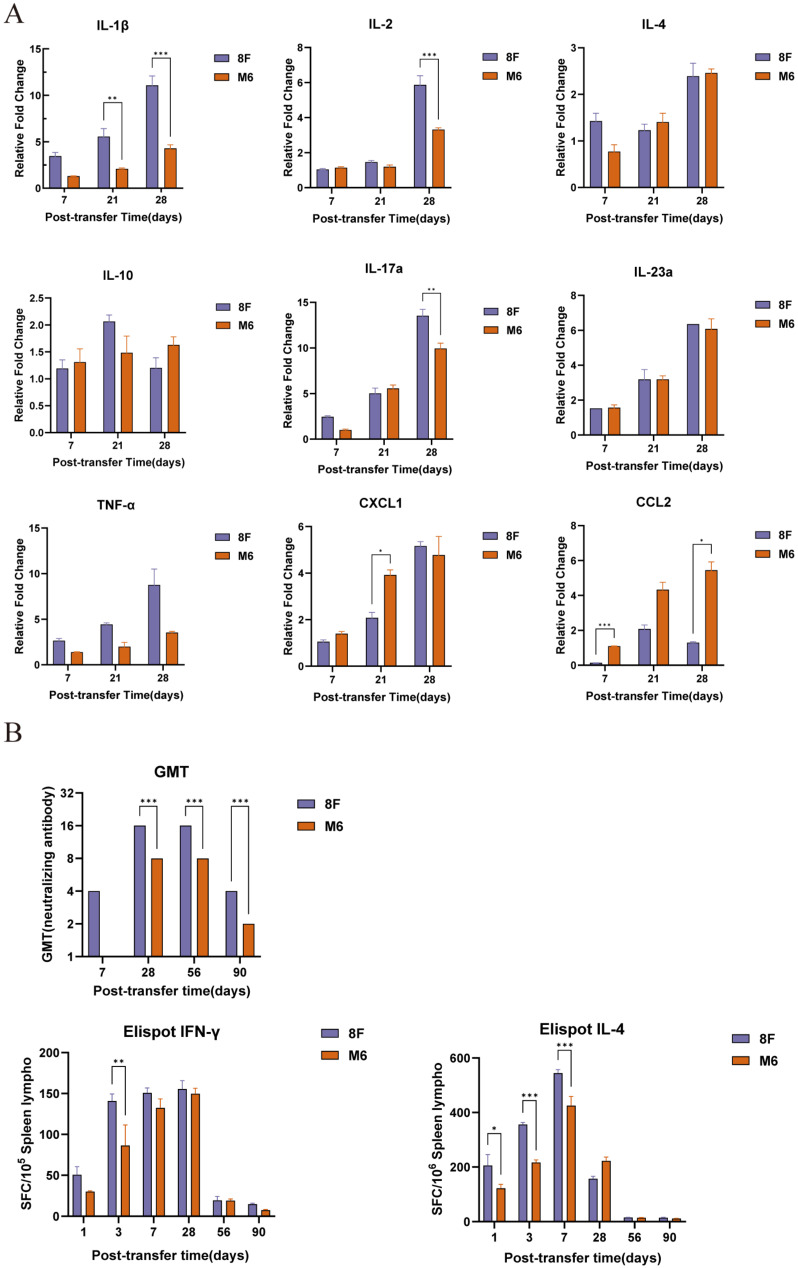
Immunological responses in mice receiving intravenous transfusion of macrophages infected with HSV-1 wild strain and attenuated strain M6. (**A**) The transcription profiles of immune signaling molecules in spleen cells at different time points post-infusion. (**B**) Changes in neutralizing antibody levels and the ELISpot responses of IFN-γ and IL-4 at different time points post-infusion. The relative expression levels of cytokines in RAW264.7 macrophages were normalized to the levels of the blank control group using the comparative Ct (ΔΔCt) method. Data are presented as the mean ± standard deviation of three independent experiments (* *p* < 0.05, ** *p* < 0.01, *** *p* < 0.001).

Our previous cytological observations indicated that the attenuated strain has a markedly different effect on macrophages compared to the wild-type strain. To further validate how this difference affects the host’s immune response, we intravenously transferred macrophages infected with the two strains back into mice and assessed the transcriptome of immune signaling molecules in the spleen cells of the mice at different time points. The results showed that, compared to the wild-type strain, macrophages infected with the attenuated strain M6 induced the relative upregulation of chemokines (such as CXCL1 and CCL2) and the significant downregulation of inflammatory molecules (such as IL-1β, TNF-α, IL-2, and IL-17a) in the reintroduced mice (Figure 4A). Meanwhile, comparing the humoral and cellular immune responses in mice re-infused with macrophages infected by the two strains, we observed that macrophages infected with the attenuated strain M6 triggered specific antibody responses and specific CTL responses similar to those induced by the wild-type strain (Figure 4B). This indicates that although infection with the attenuated strain M6 induces a lower expression of inflammatory factors, it is still capable of activating a robust immune response.

### 3.5. The Attenuated M6 Strain Induces Host-Specific Clinical Pathological Changes in Macrophages

**Figure 5:**
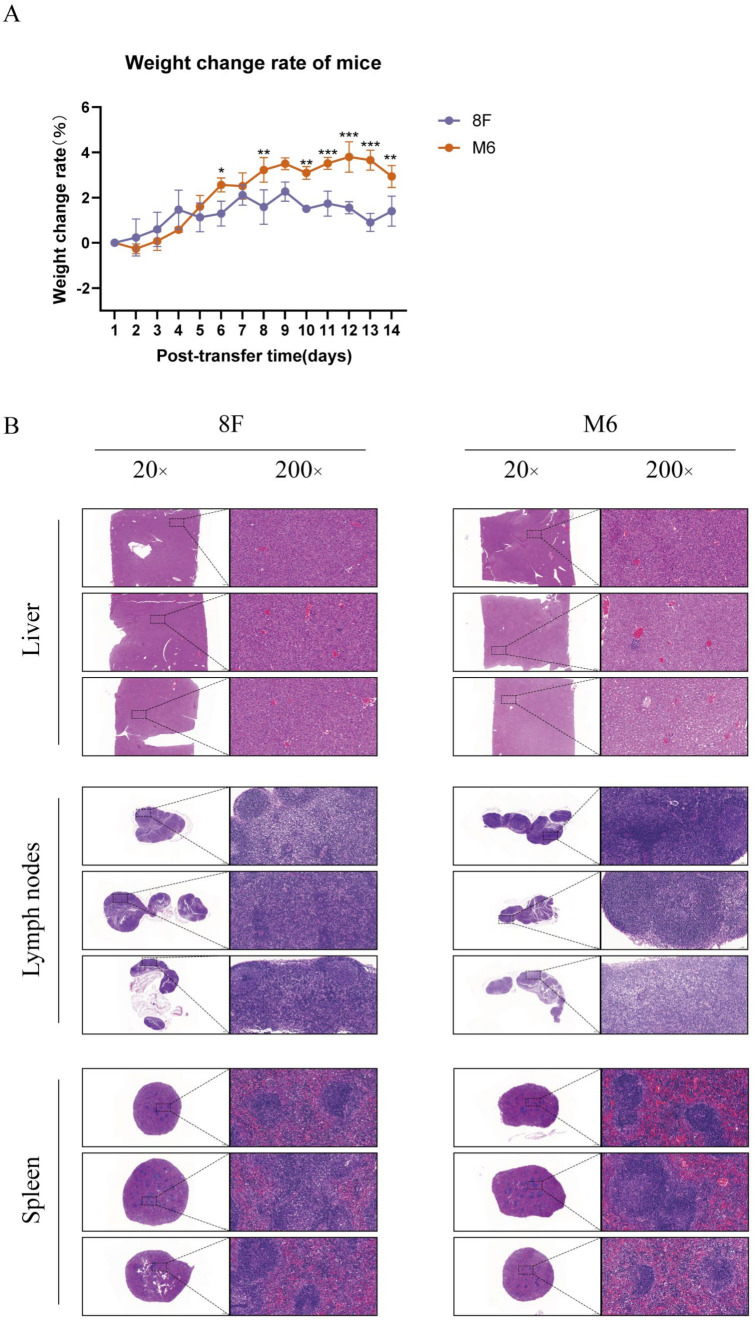
Clinical pathological observations in mice reinfused with macrophages infected by the wild-type HSV-1 and attenuated M6 strain. (**A**) Weight change rate of the two groups of mice within 14 days after tail vein reinfusion. (**B**) Pathological changes in different tissues of the two groups of mice on day 14 post tail vein reinfusion. The data are from three independent experiments that were conducted in duplicate. Statistical significance was assessed by two-way ANOVA with Bonferroni adjustment for multiple comparisons (* *p* < 0.05, ** *p* < 0.01, *** *p* < 0.001).

To investigate the pathological side effects induced by infection with the attenuated strain, we intravenously transfused macrophages infected with two different strains into mice and monitored changes in their body weight over a period of 14 days. We observed that in the wild-type strain group, the body weight change rate initially increased after transfusion but began to decline as time passed. In contrast, the body weight change rate in the attenuated strain group remained relatively stable, with a gradual upward trend (Figure 5A). Subsequently, we performed histopathological analysis on the livers, lymph nodes, and spleens of the mice on day 14 post-transfusion (Figure 5B). The results showed that in liver tissue, the wild-type strain group exhibited significant structural damage and cellular infiltration, with severe irregular structures and tissue necrosis in certain areas. In contrast, the liver structure in the attenuated strain group remained relatively intact, with localized and less severe damage. In the lymph nodes, the wild-type strain group displayed lymph node swelling and structural disarray, with areas of tissue necrosis and cellular infiltration. In the attenuated strain group, although mild swelling and some cellular infiltration were observed, the overall structure was well preserved. In spleen tissue, the wild-type strain group showed significant swelling and structural changes, accompanied by extensive cellular infiltration, while the attenuated strain group maintained relatively good structure with less damage. The body weight change rate and histopathological results suggest that macrophages infected with the wild-type strain caused significant tissue damage, leading to a higher pathological burden, whereas the macrophages infected with the attenuated M6 strain had a milder impact on the mice, showing lower immune activation and less tissue damage, indicating better safety.

## 4. Discussion

HSV-1 is a DNA virus with a complex structure, with its genome consisting of double-stranded DNA, enclosed by an icosahedral protein capsid [31,32,33]. The outer layer of the capsid is a lipid bilayer envelope on which various glycoproteins are distributed. These glycoproteins can bind to a variety of cell receptors, allowing HSV-1 to infect multiple cell types [34]. Notably, the gD glycoprotein binds to the HVEM receptor [35], enabling the virus to infect dendritic cells and macrophages, which play key roles in innate immunity [36]. This viral infection strategy, which involves binding to multiple receptors to enter cells, may trigger different immune signaling responses in various cells, especially in certain innate immune cells, thereby interfering with the normal immune response and causing associated effects [37]. At the same time, the presence of viral antigens that can bind to different cell receptors complicates the accurate assessment of host immune responses induced by these antigens, particularly neutralizing antibody responses aimed at blocking viral receptor binding, in conventional in vitro cell culture models [38]. These biological characteristics of the virus pose significant challenges in the development of vaccines. Therefore, from the current perspective of vaccine research and biological technologies, using live attenuated vaccines may be a feasible approach. Notably, the HSV-1 mutant strain M6, developed by the XU X team, exhibited significant biological differences when infecting dendritic cells compared to the wild-type virus. This suggests that the HSV-1 infection of dendritic cells, which affects the signaling from innate to adaptive immunity, is a critical aspect of the viral infection process. Furthermore, the attenuated M6 strain’s impact on antigen-presenting cells led to differences in its innate immune and pathological responses. These results support the hypothesis that the HSV-1 attenuated strain differs from the wild-type strain in its interaction with macrophages, potentially endowing it with unique immunological efficacy as an effective vaccine immunogen.

Based on this hypothesis and the infection characteristics of HSV-1 in immune cells, we first infected murine macrophage RAW264.7 cells with both the wild-type and attenuated M6 strains. The results demonstrated that HSV-1 primarily infects macrophages via the interaction between glycoprotein D (gD) and herpesvirus entry mediator (HVEM). However, when HVEM is blocked, alternative receptors on the macrophage surface, such as CD155 or Nectin-1, might facilitate viral entry. Under HVEM blockade, these alternative receptors could serve as binding targets for gD. The involvement of alternative receptors suggests that HSV-1 infection may be mediated by multiple receptors and that, under conditions of immune suppression, the virus can exploit alternative pathways to continue infecting macrophages. Additionally, we found that when macrophages were infected with the attenuated M6 strain, immune responses were likely mitigated through the reduced activation of the NF-κB signaling pathway, potentially minimizing immune-mediated tissue damage. Subsequently, we observed that the M6 strain exhibited proliferation kinetics similar to those of the wild-type virus but induced lower levels of immune signaling molecule expression. This indicates that the attenuated M6 strain may reduce immune activation by modulating the intensity of the immune response, aligning with our initial hypothesis.

Based on these cellular findings, we intravenously transfused macrophages infected with the attenuated M6 strain into mice. The results indicated that M6 infection could alleviate immune-mediated inflammatory damage. The upregulation of chemokines such as CXCL1 and CCL2 facilitated the recruitment of immune cells, potentially leading to an immune response slightly lower than that induced by the wild-type 8F strain. Finally, the M6-infected group exhibited more stable body weight changes and showed milder pathological damage in the liver, lymph nodes, and spleen, suggesting that the attenuated M6 strain has fewer immune-related adverse effects on the host.

In summary, the attenuated M6 strain is able to reduce the intensity of immune responses during macrophage infection, inducing lower levels of inflammatory cytokines while effectively activating specific antibody and CTL responses, demonstrating its potential as an effective vaccine immunogen. Further clinical pathological analysis showed that the M6 group exhibited fewer immune side effects and less tissue damage, supporting the safety and efficacy of the attenuated M6 strain as a vaccine candidate. Overall, this study suggests that the attenuated M6 strain can serve as an effective vaccine immunogen and provides new insights for future HSV-1-based vaccine research.

## Data Availability

The original contributions presented in this study are included in the article/Supplementary Materials. Further inquiries can be directed to the corresponding authors.

## Supplementary Materials

The following supporting information can be downloaded at: https://www.mdpi.com/article/10.3390/v17030392/s1, Table S1: The primer sequence of qRT-PCR.
