# Supplementary material for: Analysis of the Interaction Between the Attenuated HSV-1 Strain M6 and Macrophages Indicates Its Potential as an Effective Vaccine Immunogen
Source: Viruses. 2025 Mar 10;17(3):392. doi: 10.3390/v17030392 (PMC11945479; doi:10.3390/v17030392)
Supplement: Supplementary file 1 [file viruses-17-00392-s001.zip › viruses-3511360-supplementary.pdf]

Table S1. The primer sequence of qRT-PCR

Gene	Sequence（5'-3'）
IL-1β-F	GCAACTGTTCCTGAACTCAACT
IL-1β-R	ATCTTTTGGGGTCCGTCAACT
IL-2-F	TGAGCAGGATGGAGAATTACAGG
IL-2-R	GTCCAAGTTCATCTTCTAGGCAC
IL-4-F	GGTCTCAACCCCCAGCTAGT
IL-4-R	GCCGATGATCTCTCTCAAGTGAT
IL-10-F	GCTCTTACTGACTGGCATGAG
IL-10-R	CGCAGCTCTAGGAGCATGTG
IL-17a-F	TTTAACTCCCTTGGCGCAAAA
IL-17a-R	CTTTCCCTCCGCATTGACAC
IL-23a-F	ATGCTGGATTGCAGAGCAGTA
IL-23a-R	ACGGGGCACATTATTTTTAGTCT
CXCL-1-F	CTGGGATTCACCTCAAGAACATC
CXCL-1-R	CAGGGTCAAGGCAAGCCTC
CCL2-F	TTAAAAACCTGGATCGGAACCAA
CCL2-R	GCATTAGCTTCAGATTTACGGGT
CCL5-F	GCTGCTTTGCCTACCTCTCC
CCL5-R	TCGAGTGACAAACACGACTGC
CXCL9-F	TCCTTTTGGGCATCATCTTCC
CXCL9-R	TTTGTAGTGGATCGTGCCTCG
GAPDH-F	AGGTCGGTGTGAACGGATTTG
GAPDH-R	TGTAGACCATGTAGTTGAGGTCA
TNF-α-F	CCCTCACACTCAGATCATCTTCT
TNF-α-R	GCTACGACGTGGGCTACAG
IFN-α-F	CTTCCTCAGACTCATAACCT
IFN-α-R	AGTCCTTCCTGTCCTTCA
IFN-β-F	CAGCTCCAAGAAAGGACGAAC
IFN-β-R	GGCAGTGTAACTCTTCTGCAT

FasL F-F	TCCGTGAGTTCACCAACCAAA
FasL F-R	GGGGGTTCCCTGTTAAATGGG
TRAIL-F	ATGGTGATTTGCATAGTGCTCC
TRAIL-R	GCAAGCAGGGTCTGTTCAAGA
GM-CSF -F	GGCCTTGGAAGCATGTAGAGG
GM-CSF -R	GGAGAACTCGTTAGAGACGACTT
